# International lack of equity in modern obesity therapy: the critical need for change in health policy

**DOI:** 10.1038/s41366-022-01176-2

**Published:** 2022-07-01

**Authors:** Pia Roser, Simar S. Bajaj, Fatima Cody Stanford

**Affiliations:** 1 University Medical Center Hamburg-Eppendorf, Department of Endocrinology and Diabetes, Hamburg, Germany; 2 Harvard University, Department of the History of Science, Cambridge, MA USA; 3 Massachusetts General Hospital, MGH Weight Center, Department of Medicine-Division of Endocrinology-Neuroendocrine, Department of Pediatrics-Division of Endocrinology, Nutrition Obesity Research Center at Harvard (NORCH), Boston, MA USA

**Keywords:** Health policy, Weight management, Obesity

Patients with obesity often painfully say that “I wish I had diabetes” because health insurers cover the cost of the most effective weight-loss medications only when patients also carry a diagnosis of Type 2 Diabetes. The United Nations has launched the Sustainable Development Goals (SDGs) for 2030 to reduce premature mortality from non-communicable diseases by one-third through prevention and treatment [1]. However, the lack of equitable care across chronic disease states makes this objective unattainable. Specifically, there is substantial misalignment with health insurers’ lack of coverage of anti-obesity medications.

Obesity has become one of the leading chronic diseases in the world, with considerable societal and health-related costs [2]. Although there is evidence for the benefits of obesity treatment when provided [3], the lack of acknowledgment of obesity as a disease leads to stigmatization and a lack of treatment equality. Thomas and colleagues [4] note that although 45.9% of men and 45.0% of women in the U.S. were candidates for treatment, only 1-2% of adults with obesity received anti-obesity medication, in sharp contrast to the 8.4% of U.S. adults diagnosed with diabetes of whom 86% receive pharmacotherapy. Patients with obesity bear the consequences of their disease being poorly integrated into public healthcare.

Currently, there are several medications with a high level of efficacy for treating obesity. Semaglutide, for example, is a Glucagon-Like Peptide-1 Receptor Agonist (GLP-1 RA) that works by mimicking a hormone naturally released in the gastrointestinal tract to slow down stomach emptying and target brain regions concerning appetite reduction and food intake [5]. In June 2021 and January 2022, the FDA and the European Medicines Agency (EMA), respectively, approved Semaglutide under the name of Wegovy “for weight management in adults with a body mass index (BMI) of 27 kg/m^2^ or greater (with at least one weight-related medical condition) and patients with a BMI of 30 kg/m^2^ or greater” [6].

Unfortunately, most U.S. private health insurers refuse to cover anti-obesity therapies, requiring patients to pay out of pocket. Among 136 marketplace plans in 2018, 11% had some coverage for weight loss drugs in only nine states. Medicare also excludes anti-obesity pharmacotherapy, and Medicaid, while variable, excludes coverage in 6 states with the highest prevalence of obesity [7]. In Europe, insurance coverage for anti-obesity medications similarly varies from country to country, but in Germany, there is no indication for anti-obesity medications for body weight regulation under Social Code Book 5, Section 34 [8]. Currently, the German legislature does not differentiate between treating obesity as a chronic disease from the recreational pursuit of weight loss for vanity, thus ignoring that obesity is a complex, multifactorial disease.

But in patients with concomitant obesity and diabetes, there is, in fact, health insurance coverage for GLP-1 RAs. As such, with the cost for a pack of Wegovy reaching from $99 in the United Kingdom to $1,350 in the U.S., it has become common for patients with obesity to hope for a BMI and health status severe enough to satisfy insurance requirements. This is illogical.

The drug policies of weight-loss treatment are a critical aspect of equity. In 1971, philosopher John Rawls advocated for the betterment of the “least advantaged” in his *A Theory of Justice*. According to Rawls, the legitimacy of inequality is that the less privileged still derive benefits from it [9]. While some argue that self-funding may increase patients’ commitment to weight loss, research demonstrates that higher prices are associated with access barriers, particularly for anti-obesity medications [10]. The concern that coverage of anti-obesity pharmacotherapy diminishes the importance of lifestyle interventions is similarly unconvincing. Indeed, lifestyle interventions alone are often ineffective for patients with obesity because the biological response to restoring weight to the highest-sustained level becomes more robust as weight loss increases [11]. Obesity requires multi-modality care, including pharmacotherapy, lifestyle and behavioral modifications, and more.

Healthcare insurers exacerbate underdiagnosis of obesity in clinical practice by often refusing to cover anti-obesity medications. We suggest that passage of the Treat and Reduce Obesity Act in the U.S. and adaptation of the Social Code by the Federal Joint Committee in Germany are critical to ensure coverage and access. Although this may raise insurance premiums, the availability of semaglutide for both diabetes and obesity, but at 51% higher cost for obesity in the U.S. [12], suggests that pharmaceutical companies can charge the same lower price for both brands and absorb some of the additional costs associated with obesity coverage. Furthermore, given that overweight and obesity cost the world $990 billion per annum [13], it may be more cost-effective to cover pharmacotherapy than to allow this burden of disease to perpetuate and expand unhindered. Although prevention of obesity is necessary, only a multifaceted approach that includes access to anti-obesity medications is sufficient.

We recognize there are multiple barriers to obesity screening, diagnosis, and treatment, among which is a bias against obesity to the detriment of these patients’ health and well-being. We demand the inclusion of obesity in insurers’ drug policy plans worldwide because the status quo is insufficient for the burden of the disease, the demand for treatment, and the efficacy and safety of anti-obesity medications. Although coverage of anti-obesity drugs alone will not solve obesity, it is critical to prevent further discrimination against these patients in accordance with the 2030 SDGs for a healthier, more equitable world. Time is running short.

## References

[1] Network SDS Indicators and a Monitoring Framework Launching a data revolution for the Sustainable Development Goals. Available from: https://indicators.report/targets/3-4/.

[2] Hecker J, Freijer K, Hiligsmann M, Evers SMAA (2022). Burden of disease study of overweight and obesity; the societal impact in terms of cost-of-illness and health-related quality of life. BMC Public Health.

[3] Nielsen HJ, Nedrebø BG, Fosså A, Andersen JR, Assmus J, Dagsland VH (2022). Seven-year trajectories of body weight, quality of life and comorbidities following Roux-en-Y gastric bypass and sleeve gastrectomy. Int J Obesity.

[4] Thomas CE, Mauer EA, Shukla AP, Rathi S, Aronne LJ (2016). Low adoption of weight loss medications: A comparison of prescribing patterns of anti-obesity pharmacotherapies and SGLT2s. Obesity.

[5] Kushner RF, Calanna S, Davies M, Dicker D, Garvey WT, Goldman B, et al. Semaglutide 2.4 mg for the Treatment of Obesity: Key Elements of the STEP Trials 1 to 5. Obesity (Silver Spring). 2020;28(6):1050–61. 10.1002/oby.22794 PMC7318657 32441473

[6] Agency EM Wegovy 2022. Available from: https://www.ema.europa.eu/en/medicines/human/EPAR/wegovy.

[7] Gomez G, Stanford FCUS (2018). health policy and prescription drug coverage of FDA-approved medications for the treatment of obesity. Int J Obes.

[8] Justice TFMo. Social Code Book V - Statutory Health Insurance - (Article 1 of the Act of December 20, 1988, BGBl. I S. 2477) § 34 Excluded drugs, remedies and aids. Available from: https://www.gesetze-im.internet.de/sgb_5/__34.html.

[9] Wenar L John RawlsSummer 2021.

[10] Morgan SG, Lee A (2017). Cost-related non-adherence to prescribed medicines among older adults: a cross-sectional analysis of a survey in 11 developed countries. BMJ Open.

[11] Rosenbaum M, Leibel RL. Adaptive thermogenesis in humans. Int J Obes. 2010;34 Suppl 1 (0 1)):S47–55. 10.1038/ijo.2010.184 PMC3673773 20935667

[12] Kolata G The Doctor Prescribed an Obesity Drug. Her Insurer Called It ‘Vanity.’ The New York Times 2022. Available from: https://www.nytimes.com/2022/05/31/health/obesity-drugs.insurance.html?referringSource=articleShare.

[13] All countries significantly off track to meet 2025 WHO targets on Obesity [press release]. World Obesity Federation 2020.

